# VprBP directs epigenetic gene silencing through histone H2A phosphorylation in colon cancer

**DOI:** 10.1002/1878-0261.13068

**Published:** 2021-08-08

**Authors:** Nikhil Baban Ghate, Sangnam Kim, Erin Spiller, Sungmin Kim, Yonghwan Shin, Suhn K. Rhie, Goar Smbatyan, Heinz‐Josef Lenz, Shannon M. Mumenthaler, Woojin An

**Affiliations:** ^1^ Department of Biochemistry and Molecular Medicine Norris Comprehensive Cancer Center University of Southern California Los Angeles CA USA; ^2^ Lawrence J. Ellison Institute for Transformative Medicine University of Southern California Los Angeles CA USA; ^3^ Division of Medical Oncology Norris Comprehensive Cancer Center University of Southern California Los Angeles CA USA

**Keywords:** chromatin, colon cancer, H2A, phosphorylation, transcription, VprBP

## Abstract

Histone modification is aberrantly regulated in cancer and generates an unbalanced state of gene transcription. VprBP, a recently identified kinase, phosphorylates histone H2A on threonine 120 (T120) and is involved in oncogenic transcriptional dysregulation; however, its specific role in colon cancer is undefined. Here, we show that VprBP is overexpressed in colon cancer and directly contributes to epigenetic gene silencing and cancer pathogenesis. Mechanistically, the observed function of VprBP is mediated through H2AT120 phosphorylation (H2AT120p)‐driven transcriptional repression of growth regulatory genes, resulting in a significantly higher proliferative capacity of colon cancer cells. Our preclinical studies using organoid and xenograft models demonstrate that treatment with the VprBP inhibitor B32B3 impairs colonic tumor growth by blocking H2AT120p and reactivating a transcriptional program resembling that of normal cells. Collectively, our work describes VprBP as a master kinase contributing to the development and progression of colon cancer, making it a new molecular target for novel therapeutic strategies.

## Graphical abstract

VprBP is a recently identified kinase that induces transcriptional silencing of growth suppressive genes to cause cancer. In this study, we demonstrate that VprBP is overexpressed in colon cancer and drives tumorigenic transcription program through H2AT120 phosphorylation. VprBP‐mediated H2AT120 phosphorylation is functionally important, because blocking VprBP kinase activity impedes colonic tumor growth.

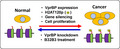

**Abbreviations**
ChIP: chromatin immunoprecipitation
ChIP‐qPCR: ChIP‐quantitative polymerase chain reaction
COAD: colon adenocarcinoma
CR: coding region
EGFP: enhanced‐green fluorescent protein
FBS: fetal bovine serum
FDR: false discovery rate
GEPIA: gene expression profiling interactive analysis
HCS: high content screening
IRB: Institutional Review Board
IVIS: *in vivo* imaging system
OS: overall survival
PCA: principal component analysis
PDOs: patient‐derived organoids
PR: promoter region
RNA‐seq: RNA sequencing
RT‐qPCR: real‐time‐quantitative polymerase chain reaction
TCGA: The Cancer Genome Atlas
TSS: transcription start site

## 1 Introduction

Eukaryotic genomic DNA is hierarchically packaged by histone proteins to form a highly organized structure of chromatin. This chromatin organization and its dynamic nature provide the functional flexibility for the regulation of various genome functions including gene transcription in the nucleus. Among several known processes that affect chromatin dynamics, covalent post‐translational modifications of histone proteins are known to play major roles in governing chromatin‐dependent activation or repression of transcription [1]. While the biochemical and molecular mechanisms are still not well understood, a growing body of evidence indicates that histone modifications generate distinct changes in chromatin structure and facilitate the recruitment of transcriptional regulators to particular genomic loci [2, 3, 4]. Since establishing a correct state of histone modification is of vital importance for ensuring proper gene transcription program, all these epigenetic processes are tightly regulated. The dysregulation of histone‐modifying factors by mutations or overexpression can trigger abnormal pattern of histone modifications and disrupt the epigenetic balance of gene expression, thereby act as an oncogenic driver in human cancers [5].

VprBP was originally identified as a protein that can interact with the HIV‐1 accessory protein viral protein R (Vpr) and hence was named Vpr‐binding protein (VprBP) [6]. Since its discovery, VprBP (also called DCAF‐1) has been mainly characterized as a substrate receptor acting in the DDB1‐Cul4‐ROC1 E3 ubiquitin ligase complex and controlling cell development [7, 8, 9]. Unexpectedly, however, we recently discovered an additional role for VprBP as an effector that binds histone H3 tails protruding from promoter nucleosomes and inactivates chromatin transcription [10]. More relevant to the current study, our continued investigation revealed that VprBP is highly expressed in cancer cells and has an intrinsic kinase activity capable of phosphorylating histone H2A at T120 [11]. H2A T120 phosphorylation (H2AT120p) is necessary for VprBP transrepression activity, because VprBP kinase‐dead mutation (K194R) and H2AT120p blocking mutation (T120A) eliminate the ability of VprBP to inactivate gene transcription in our functional assays. Moreover, results from our gene expression profiling strongly suggested that targeting and silencing growth regulatory genes reflects the role of VprBP in tumorigenesis. Our immunohistochemical analysis of commercial tissue microarrays also established a link between elevated expression of VprBP and increased levels of H2AT120p in bladder, breast, and prostate cancer cells. Consistent with these data, knockdown of VprBP decreased H2AT120p and impaired prostate cancer cell proliferation and xenograft tumor growth. Given that VprBP‐mediated H2AT120p plays a causal role in tumorigenesis, we also have identified a small‐molecule inhibitor, named B32B3, capable of inhibiting VprBP kinase activity and tumor growth, even causing some partial tumor regression, in prostate xenograft models [11]. These data provide a mechanism to account for VprBP function in establishing inactive chromatin states and inducing abnormal gene silencing in prostate cancer cells. Whether VprBP participates in the development of other types of cancer is currently unknown, but detection of H2AT120p among different cell types allows the assumption that VprBP‐mediated H2AT120p might exert its role in driving oncogenic transformation of other types of cancer cells.

Colon cancer is a leading cause of cancer deaths in the world and has been considered to arise from genetic alterations in genes encoding factors that help control cell growth and proliferation [12, 13]. However, accumulating evidence indicates that epigenetic alterations generated by dysregulation of epigenetic modifiers play pivotal roles in the initiation, promotion, and progression of colon cancer [14, 15, 16, 17]. Especially, histone modification is increasingly recognized as an essential process to control oncogenic transcription program and influence the occurrence and development of colon cancer [18, 19, 20, 21, 22, 23, 24, 25]. The detailed mechanisms to link histone modification to altering gene expression and promoting colonic carcinogenesis, however, remain unclear. In the present study, we employed a combination of cell lines, xenograft models, and tumor organoids to dissect the functional contribution of VprBP to the development and progression of colon cancer. Our data indicate that VprBP localizes at genes regulating cell growth and establishes transcriptional silencing in colon cancer cells. VprBP executes these functions in a manner dependent on H2AT120p, as kinase‐dead mutation almost completely abolishes the gene silencing potential of VprBP. Moreover, chemical inhibition of VprBP leads to target gene reactivation and tumor growth suppression, supporting the role of VprBP as a promising therapeutic target in colon cancer.

## 2 Materials and methods

### 2.1 Cell lines, constructs, and antibodies

HCT116, HCT15, HT29, SW620, SW480, SW1222, RKO, Caco2, LS174T, and T84 cells were cultured in Dulbecco’s modified Eagle’s medium containing 10% fetal bovine serum (FBS). LOVO and NCM460 cells were cultured in RPMI1640 medium supplemented with 10% FBS. For mammalian expression of VprBP, the VprBP cDNA was amplified by PCR and ligated into the lentiviral expression vector pLenti‐Hygro (Addgene, Watertown, MA, USA) containing 5’ 3×FLAG coding sequence. To generate VprBP K194R expression vector, wild‐type VprBP cDNA was mutated by using Q5 Site‐Directed Mutagenesis Kit (New England Biolabs, Ipswich, MA, USA) after the construction. Antibodies used in this study are as follows: anti‐actin and anti‐FLAG antibodies from Sigma‐Aldrich, St. Louis, MO, USA; anti‐Histone H2AT120 phospho antibody from Active Motif, Carlsbad, CA, USA; anti‐VprBP antibody from Proteintech, Rosemont, IL, USA; and anti‐Histone H2A antibody from Abcam, Cambridge, MA, USA.

### 2.2 Tissue samples

A total of 20 tissue samples including 10 colon cancer tissues and 10 normal tissue counterparts were obtained from the Division of Medical Oncology, Norris Comprehensive Cancer Center at University of Southern California, USA. The study was approved by University of Southern California’s Ethics Committee, and all patients provided written informed consent according to the committee’s regulations. The study methodologies were conformed to the standards set by the Declaration of Helsinki.

### 2.3 Protein and histone extraction and western blotting

Whole cell lysates were prepared from SW620 and Caco2 cells using M‐PER™ Mammalian Protein Extraction Reagent (Thermo Scientific, Waltham, MA, USA) according to the manufacturer’s instructions. For protein extraction from patient tissue samples, tissues which had been flash‐frozen in liquid nitrogen were ground with a chilled mortar and pestle. Then, T‐PER™ Tissue Protein Extraction Reagent (Thermo Scientific) was added to the finely grounded tissue, and tissue lysates were prepared according to the manufacturer’s protocol. Total histone proteins were acid‐extracted from the cultured cells and patient tissue samples as described [26].

### 2.4 RNA interference

DNA oligonucleotides (5’‐CGAGAAACTGAGTCAAATGAA‐3’) encoding shRNAs specific for VprBP coding region were annealed and ligated into the lentiviral expression vector pLKO.1 (Addgene). Lentiviral particles were generated in 293T cells by transfecting plasmids encoding VSV‐G, NL‐BH, and the shRNA. Two days after transfection, the soups containing the viruses were collected and used to infect SW620 and Caco2 cells in the presence of polybrene (8 μg·mL^−1^). The cell lines were selected for two weeks in the presence of puromycin (2 μg·mL^−1^). For rescue experiments, VprBP‐depleted cells were infected with lentiviruses expressing shRNA‐resistant VprBP wild‐type or kinase‐dead mutant K194R and selected for two weeks in the presence of hygromycin (800 μg·mL^−1^ for SW620 cells and 200 μg·mL^−1^ for Caco2 cells).

### 2.5 RNA‐seq

RNA was prepared from SW620 cells using the Qiagen RNeasy kit (Qiagen Inc., Valencia, CA, USA) according to the manufacturer’s instructions. After quality control, strand‐specific libraries were prepared using a KAPA Stranded mRNA‐Seq Kit with KAPA mRNA Capture Beads (Kapa Biosystems Inc., Wilmington, MA, USA) and validated on an Agilent Bioanalyzer with the DNA1000 kit. Pooled libraries were prepared, denatured, diluted to 15 pM, and then clonally clustered onto the sequencing flow cell using the Illumina cBOT Cluster Generation Station and Cluster Kit v3‐cBot‐HS. The clustered flow cell was sequenced with 1X50 SE reads on the Illumina HiSeq 2000 according to the manufacturer’s protocol. Base conversion was made using olb version 1.9, demultiplexed, and converted to Fastq using CASAVA version 1.8 (Illumina, San Diego, CA, USA). The library preparation and sequencing were done as described [27]. Sequenced RNA‐seq reads were mapped to hg38 gencode version 29 [28] using star 2.6.1d [29]. Aligned reads were quantified at gene levels, and gene counts were normalized using the upper quartile normalization method. After principal component analysis with normalized gene counts, differentially expressed genes were selected by using the Gene Specific Algorithm from Partek^®^ Flow^®^ software (Partek Inc., Chesterfield, MO, USA). Fold change and false discovery rate of genes were used to generate a volcano plot. We used a false discovery rate cutoff of 0.05 and absolute fold change > 2 to select statistically significantly differentially expressed genes. Gene ontology analysis of differentially expressed genes was performed using Ingenuity Pathway Analysis tool (IPA^®^ version 52912811) (Qiagen Inc.). The heatmap was generated by calculating *Z* score of gene expression levels using the Generalized Minimum Distance r package heatmap.3 function [30].

### 2.6 RT‐qPCR

Total RNA was isolated from cells using the RNeasy Mini kit (Qiagen Inc.) and converted to first‐strand cDNA using the SuperScript III First‐Strand System Kit (Thermo Scientific). Real‐time RT‐qPCR was carried out with SYBR Green Real‐time PCR Master Mixes (Thermo Scientific) according to the manufacturer’s protocol. The primers used for RT‐qPCR are listed in Table 1. All mRNA values were normalized to β‐actin mRNA levels. All reactions were run in triplicate, and results were averaged.

**Table 1:** List of the primers used in RT‐qPCR.

Gene name	Forward (5’‐3’)	Reverse primer (5’‐3’)
NCAM1	AGAAGCAAGAGACTCTGGATGG	CCCTGTAGCTTTGGGGCATA
KNDC1	GATTGTGACCAGCCACACCT	GAATTCTCCAGGCAGGGGAC
TRIM67	CAGGTGTCTCAGGAGCAGTG	TGAAGTCCAGCTGGTGGATG
RASD1	TCATCCTGGTGTTCAGTCTGG	CGTCCACGTTCTCCTTGGTT
ZBTB32	TATGCGTGCTCTGTCTGTGG	CTTGGTCATGGCCGAGAAGT
CNGB1	CCAGAACCGAATCCTGAGGAG	GTGGAAGTAAGGGCAGCCT
ZNF154	TTACAACTGTGGAGAACACACAA	TGCTGAATGAGACTAGAGCTTTG
DCDC2B	GCCGCTGGATTTGAACGATTC	ACACAAGTGGCGAGTCACG
RPL23	GTGAAGGGGATCAAGGGACG	GTCGAATGACCACTGCTGGA
β‐actin	GTGGGGCGCCCCAGGCACCA	CTCCTTAATGTCACGCACGATTTC

### 2.7 ChIP

ChIP assays with SW620 and Caco2 cells were performed using the ChIP Assay Kit (Millipore, Burlington, MA, USA) as recently described [31]. After reversing protein–DNA crosslinks, immunoprecipitated DNA was purified and analyzed by qPCR using the primers that amplify the promoter (PR), transcription start site (TSS), and coding (CR) region of RASD1 and ZBTB32. The primers used for qPCR are listed in Table 2. Specificity of amplification was determined by melting curve analysis, and all samples were run in triplicate.

**Table 2:** List of the primers used in ChIP‐qPCR.

Gene name	Forward (5’‐3’)	Reverse primer (5’‐3’)
RASD1 (PR)	CTATTCTCTGGGGAAACTGAG	CCTCTGCGACTTCGGG
RASD1 (TSS)	AGCAGGCTGCATATAAGGG	TCCCAGGATCTGGGCAC
RASD1 (CR)	AGACGTTTTCATCCTGGTGT	TTCTTGAGGCAAGACTTGGT
ZBTB32 (PR)	CCTGAGCGGGAGAATGC	CCGTTAAAAGTGGGGAATGG
ZBTB32 (TSS)	CACTTTTAACGGTCACTCGG	AAAAACTATGGCACTCACCG
ZBTB32 (CR)	ACAGGGCTAAAAAGCCAGAT	CATCTCCTGTTCTCTCCCAC
RPL23	ACGGGTCAATAAATAGAGCAATACT	ATACAGTTGAGAAGTGCTGCAGAT
RPL23	TAATAAGGCAGCGCCCAGAG	CTTCGACATCTTGAACGCCG
RPL23	TACTGATGGAACGGCCTGATG	CCTTGATCCCCTTCACGGAG

### 2.8 Cell viability and colony formation assays

VprBP‐depleted/rescued SW620 and Caco2 cells were seeded into 96‐well plates at a density of 2000 cells per well. After 72 h of culture, their viability was assessed by using the cell proliferation reagent WST‐1 (Roche Diagnostics, Basel, Switzerland) according to the manufacturer’s protocol. To determine the effects of B32B3, WST‐1 assays were also conducted after treating SW620 and Caco2 cells with increasing concentrations (0.05, 0.1, 0.3, 1.5, 3, and 5 µm) of B32B3 for 72 h. For colony formation assays, SW620 and Caco2 cells were seeded at a density of 1000 cells/well in 6‐well plate and treated with different concentrations of B32B3 for 72 h. Then, fresh medium is added, and cells were allowed to form colonies for additional 2 weeks. The colonies in each well were stained with 0.5% crystal violet in 20% ethanol and photographed. The colonies in each well were counted using ImageJ 1.45s software (U. S. National Institutes of Health, Bethesda, MD, USA). All assays were run in triplicate, and results presented are the average of three individual experiments.

### 2.9 Patient‐derived organoid

Tumor tissue was received from consented patients following Institutional Review Board (IRB) approval at the Norris Comprehensive Cancer Center of USC, Los Angeles CA. Cells were isolated from colon cancer tissue specimen and used to establish patient‐derived organoids (PDOs) as previously described [32]. For inhibitor treatment, PDOs were filtered through a 40 μm strainer to remove aggregates, seeded on 96‐well white walled plates at a concentration of 2.5 × 10^6^ cells per mL, and topped with 100 μL of organoid culture medium. PDOs were then incubated for 4 days and treated with B32B3 at different concentrations for 5 days. At the end of the treatment, 100 μL of CellTiter‐Glo 3D (Promega, Madison, WI, USA) was added to each well, and their luminescence was measured in a luminometer following the manufacturer’s protocol. Images were also acquired at 0, 3, and 5 days postdrug treatments in brightfield, with 23 z‐stacks ranging from 20 to 460 µm at increments of 20 µm on the Operetta high content screening (HCS) platform (PerkinElmer, Waltham, MA, USA).

### 2.10 Mice xenograft

All animal experiments were performed according to protocols approved by the Institutional Animal Care and Use Committee. *In vivo* experiments were performed using Athymic Male Nude mice [(Crl:NU(NCr)‐*Foxn1^nu^*] (Charles river, Wilmington, MA, USA). The animals were acclimatized under a constant light/dark cycle at 22 ± 2 °C (12‐h light/dark cycles). All the animals were served with a general laboratory diet and water *ad libitum*. Experimental animals were attended to every six hours during the treatment period, and it was observed that there was no undesirable animal death. SW620 colon cancer cells were stably transfected with EGFP and subcutaneously injected into both right and left hind legs of mice. Mice were treated with intraperitoneal injections of vehicle (DMSO) or B32B3 at a dose of 5 mg·kg^−1^ every three days for 21 days. Fluorescent signals from the tumors were specifically detected at a wavelength of 581 nm with an *in vivo* imaging system (IVIS) after 1, 11, and 21 days of treatments. The mice were sacrificed by asphyxiation with CO_2_, and tumors were excised and weighed 21 days after treatment.

### 2.11 Immunostaining

To assess changes in H2AT120p levels, formalin‐fixed and paraffin‐embedded sections (5 mm) from SW620 tumor xenografts were subject to immunohistochemical analysis by treating with blocking reagent (50 mm Tris/HCl, pH 7.5, 150 mm NaCl, 0.3% Triton X‐100, and 5% normal goat serum) for 30 min at room temperature and incubating with H2AT120p antibody overnight at 4 °C. FITC‐tagged secondary antibody was used to detect the H2AT120p antibody in our assays. For immunofluorescence of SW620 and Caco2 cells, cells were fixed with 4% paraformaldehyde for 15 min. The corresponding samples were permeabilized with 0.3% Triton X‐100 for 10 min and immunostained with H2AT120p antibody as described previously [11].

### 2.12 Acquisition of TCGA data

The TCGA data were acquired from UCSC Xena (https://xena.ucsc.edu/) [33].

### 2.13 Statistical analysis

The statistical analysis was performed by kyplot version 6.0 (KyensLab Inc., Tokyo, Japan). The IC50 values were calculated by the formula, *Y* = 100*A1/(*X* + *A*1) where *A*1 = IC50, *Y* = response (*Y* = 100% when *X* = 0), X = inhibitory concentration. The IC50 values were compared by paired *t*‐test. To determine significance, paired *t*‐test was used. *P* < 0.05 was considered significant.

## 3 Results

### 3.1 VprBP overexpression and H2AT120 hyperphosphorylation are common in colon cancer

**Fig. 1:**
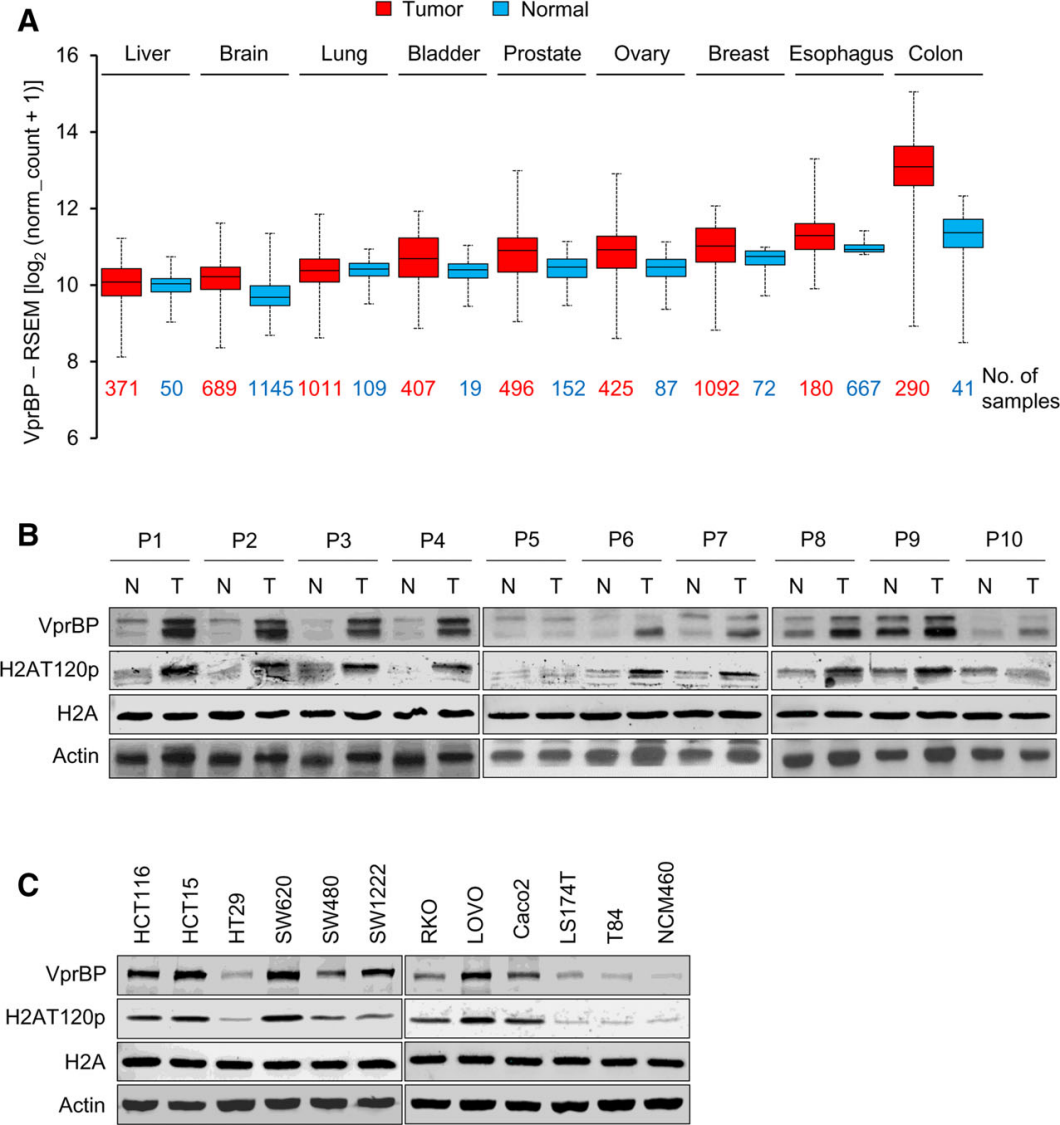
High levels of VprBP expression and H2AT120 phosphorylation in colon cancer. (A) Box and whisker plots showing the altered expression of VprBP in different cancer types in TCGA database. *X*‐axis of the plot represents normal vs cancer group, while *y*‐axis represents mRNA expression in log2 median/mean centered intensity. Boxes represent 25–75% of values, and black lines in boxes represent median expression values. The numbers of normal (blue) and tumor (red) samples analyzed are shown at the bottom. (B) Whole cell lysates and chromatin were prepared from ten human colon tumor and their adjacent normal tissue samples and analyzed by western blotting with VprBP, H2AT120p, and H2A antibodies. Actin served as a control for equal protein loading. (C) Western blot analysis of cell lysates and chromatin prepared from the indicated cell lines with VprBP, H2AT120p, and H2A antibodies. Actin served as a control for equal protein loading.

Using tumor tissue microarrays in combination with immunohistochemistry, we have previously shown that VprBP is highly expressed in tumor samples, especially in prostate, bladder, and breast tumor tissues [11]. As the tissue microarrays used in this study represent only a limited number of cancer cases, we wanted to re‐characterize VprBP expression patterns in each tumor context. Toward this end, we analyzed VprBP expression in twenty different types of cancer by using The Cancer Genome Atlas (TCGA) database. In accordance with our previous study, the TCGA analysis detected the elevated expression of VprBP in several cancer types including prostate, bladder, and breast cancers (Fig. 1A). Somewhat surprisingly, the analysis also led us to discover that VprBP overexpression is significantly more common in colon cancer as compared to other types of cancer (Fig. 1A). This observation is in good agreement with our western blot results showing that VprBP protein levels are higher in 8 out of 10 colonic tumor tissue samples when compared to their adjacent normal tissues. Given the demonstrated role of VprBP in H2AT120p in prostate cancer cells, we also wondered whether H2AT120p levels are altered in colonic tumor samples. Indeed, our western blot analysis detected high levels of H2AT120p in all the tumor samples showing VprBP overexpression (Fig. 1B). In additional investigation of VprBP pathogenicity in colon cancer patients, overall survival (OS) was evaluated in a large public clinical database which integrates gene expression and patient survival. When we conducted Kaplan–Meier survival analysis and statistically compared using the log‐rank test, somewhat uneven distribution of cases between the two survival curves generated a *P*‐value of 0.788 (Fig. S1A). Nevertheless, the OS of high VprBP expression group was found to be lower than that of low VprBP expression group, and this observation is consistent with the results obtained from colonic tumor samples (Fig. 1B). In analyzing VprBP expression levels in four stages of colon cancer, overexpression of VprBP was detected in the four stages of colon cancer with only minor variations (Fig. S1B). To provide further evidence for VprBP overexpression in colon cancer, we used 11 colon cancer and 1 normal (NCM460) cell lines for our western blot analysis. In most of the cancer cell lines examined, higher levels of VprBP expression and increased levels of H2AT120p were clearly detected (Fig. 1C). Together, these initial observations link VprBP and H2AT120p to colonic tumorigenesis and provide a rationale to focus on their potential role in mediating oncogenic gene silencing in colon cancer.

### 3.2 VprBP is the kinase responsible for H2AT120 phosphorylation in colon cancer cells

**Fig. 2:**
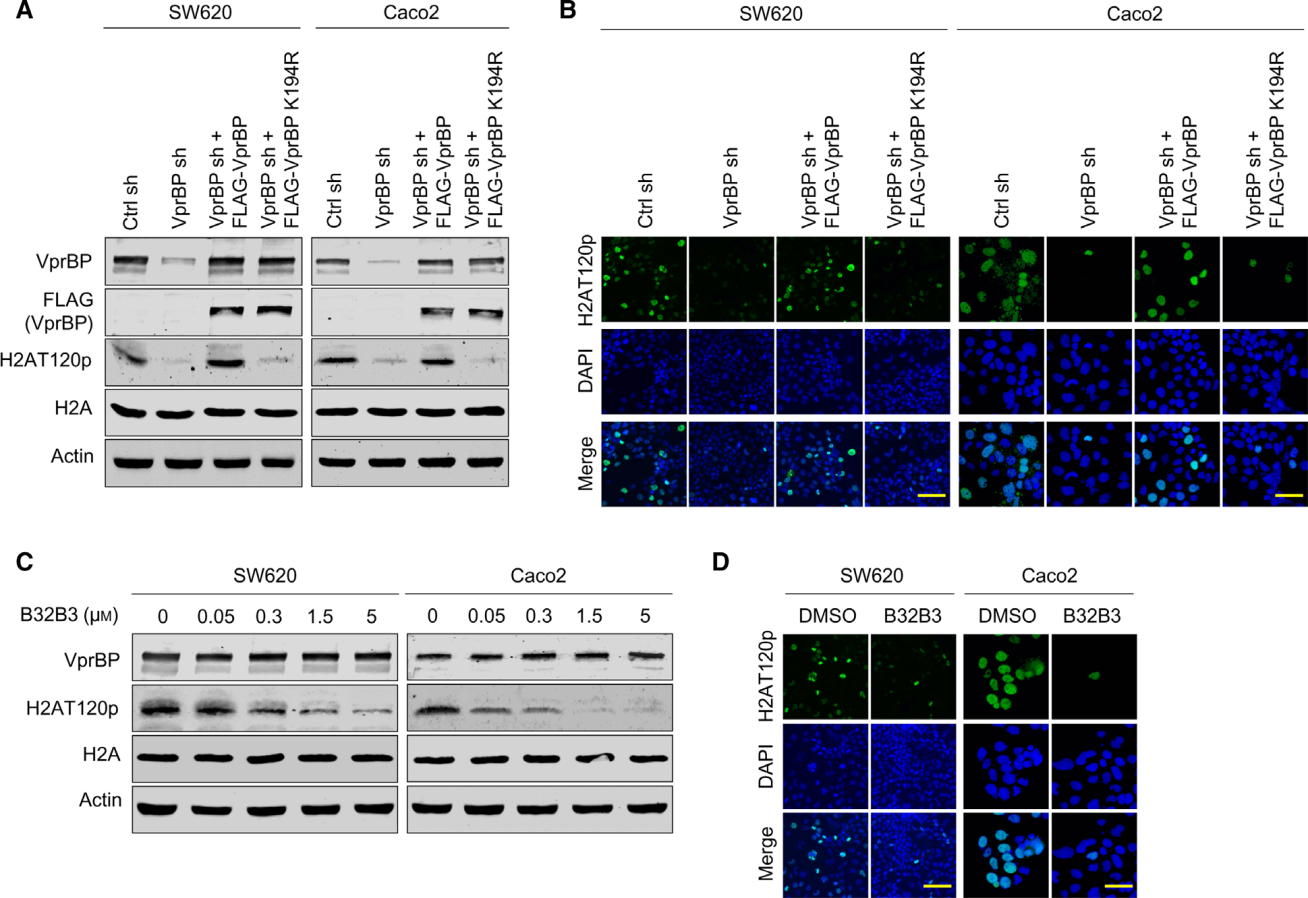
VprBP mediates H2AT120 phosphorylation in colon cancer cells. (A) SW620 and Caco2 colon cancer cells were transfected with nontargeting control (Ctrl) or VprBP shRNA, and whole cell lysates and chromatin fractions were analyzed by western blotting with the indicated antibodies. VprBP‐depleted cells were also complemented by shRNA‐resistant VprBP wild‐type or kinase‐dead mutant K194R to check their rescue effects. Actin was used as a loading control. Shown are the representative results of three independent immunoblot experiments. (B) VprBP‐depleted SW620 and Caco2 cells were transfected with VprBP wild‐type or K194R as in (A) and immunostained with H2AT120p antibody. Scale bars correspond to 10 µm. Representative images of stained cells are shown. Images are representative of three independent experiments. (C) SW620 and Caco2 cells were grown in the presence of the indicated concentrations of B32B3 for 72 h. Cell lysates and chromatin fractions were prepared and probed for VprBP, H2AT120p, and H2A by western blot. Shown are the representative results of three independent experiments. (D) After treating with B32B3 as in (C), SW620 and Caco2 cells were immunostained with H2AT120p antibody to monitor relative changes in H2AT120p levels. Images are representative of three independent experiments.

Because VprBP has been reported as a major kinase for H2AT120p in prostate cancer cells [11], the positive correlation between VprBP expression and H2AT120p observed in Fig. 1 encourages the possibility that VprBP is responsible for H2AT120p in colon cancer cells. To test this possibility, we depleted VprBP in two colon cancer cells SW620 and Caco2 showing high levels of VprBP expression and H2AT120p. In this study, it was important that VprBP is continuously depleted, as this allows all our investigations under identical conditions. This was achieved by using a lentiviral shRNA infection system. Western blot analysis confirmed that the stable transfection of VprBP shRNA efficiently silenced the expression of VprBP in SW620 and Caco2 cells. As shown in Fig. 2A,B, our initial western blotting and immunostaining clearly showed that knockdown of VprBP decreased H2AT120p in SW620 and Caco2 cells. Ectopic expression of VprBP wild‐type, but not VprBP K194R kinase‐dead mutant, in VprBP‐depleted cells restored H2AT120p to levels quantitatively similar to those observed with mock‐depleted control cells—underscoring the importance of VprBP kinase activity for H2AT120p in colon cancer cells.

We previously identified B32B3 as an inhibitor of VprBP through high‐throughput screening of small‐molecule libraries and demonstrated that B32B3 can selectively inhibit VprBP kinase activity toward H2AT120 [11]. We therefore treated SW620 and Caco2 cells with B32B3 and evaluated changes in H2AT120p. The range of B32B3 concentrations used is similar to those reported previously to result in on‐target inhibition of VprBP kinase activity, including inhibition of H2AT120p, in prostate cancer cells. When SW620 and Caco2 cells were exposed to five different concentrations (0, 0.05, 0.3, 1.5, and 5 μm) of B32B3, we found that B32B3 potently blocked H2AT120p with a half‐maximal inhibitory concentration (IC50) of ˜ 0.3 µm (Fig. 2C). Consistent with these results, our immunostaining of SW620 and Caco2 cells after B32B3 treatment also detected a significant reduction in H2AT120p—indicative of efficient blockage of VprBP kinase activity by B32B3 (Fig. 2D). Collectively, these observations are in consonance with our knockdown experiment data and underscore the notion that VprBP is responsible for the H2AT120p event in colon cancer cells.

### 3.3 VprBP inactivates growth regulatory genes via H2AT120 phosphorylation

**Fig. 3:**
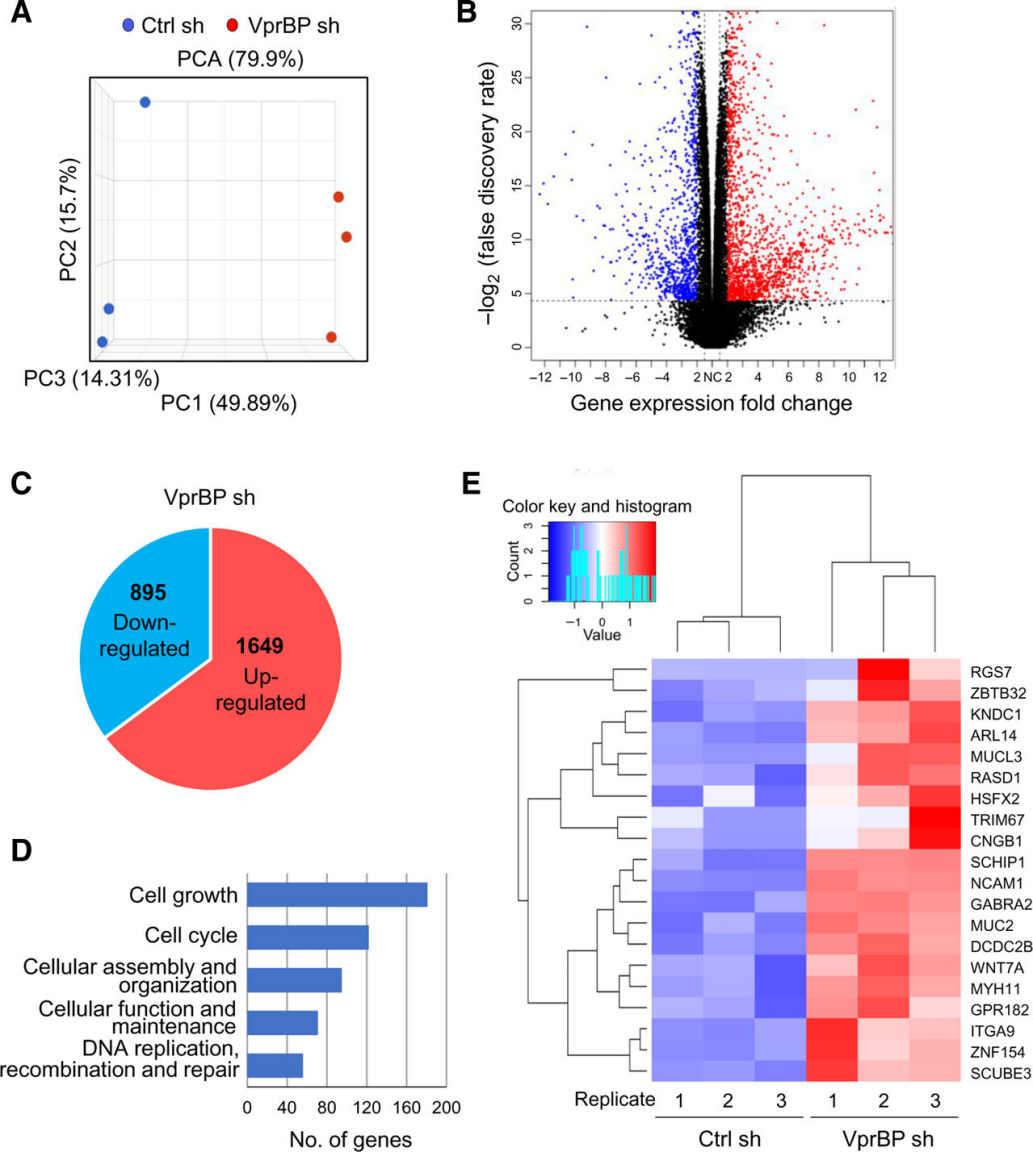
VprBP impairs the expression of growth‐controlling genes. (A) Principal component analysis (PCA) results of RNA‐seq datasets generated in SW620 cells. VprBP knockdown (VprBP sh) group is shown in red, and control (Ctrl sh) group is shown in blue. Three replicates are generated per group. (B) A volcano plot of RNA‐seq datasets is shown. ‐log2 (false discovery rate, FDR) is shown on the y‐axis, and fold change of gene expression between VprBP knockdown and control groups is shown on the x‐axis. Genes modulated after VprBP depletion are colored in blue (downregulated) and red (upregulated). (C) A pie chart shows 1649 upregulated genes and 895 downregulated genes. Genes whose expression levels are changed more than 2‐fold in response to VprBP depletion are selected (FDR < 0.05). (D) Gene ontology analysis of the activated genes after knockdown of VprBP using Ingenuity Pathway Analysis (IPA^®^ version 52912811) tool developed by Qiagen. (E) A heatmap shows example 20 genes activated upon VprBP depletion. Normalized gene expression levels (*Z*‐scores) are plotted. Color key indicates for *Z* score (blue: low expression and red: high expression). Histogram indicates the number of genes that belong to each category.

We have previously reported that VprBP is overexpressed and catalyzes H2AT120p to negatively regulate tumor suppressor genes in prostate cancer cells [11]. This result and our demonstration of VprBP overexpression in colon cancer cells suggested the possibility that VprBP might likewise function in colon cancer cells. To test this, we isolated total RNA from control or VprBP‐depleted SW620 colon cancer cells and conducted RNA sequencing (RNA‐seq). In the principal component analysis of RNA‐seq data, samples for each group were found to be markedly separated from each other, but close clustering of replicates from groups indicated minimal variability in the quality of analyzed replicates (Fig. 3A). As can be seen in Fig. 3B,C and S2, our comparative transcriptome analysis with a fold change cutoff of 2 identified a total of 2544 genes differentially expressed in response to VprBP knockdown. In more detailed examination, we found that 65% (1649) of these genes were upregulated at least 2‐fold in VprBP‐depleted cells, supporting a major function for VprBP in gene silencing (Fig. 3C). Consistent with our recent publication implicating VprBP in prostate cancer, gene ontology analysis also revealed that many of the activated genes encode key components of anti‐oncogenic growth regulatory pathways, including those known to suppress cancer initiation and progression (Fig. 3D). Another evidence supporting the role of VprBP in colonic tumorigenesis came from the fact that our analysis of the leading‐edge subset in gene set identified 20 genes regulating cell growth and proliferation (Fig. 3E).

**Fig. 4:**
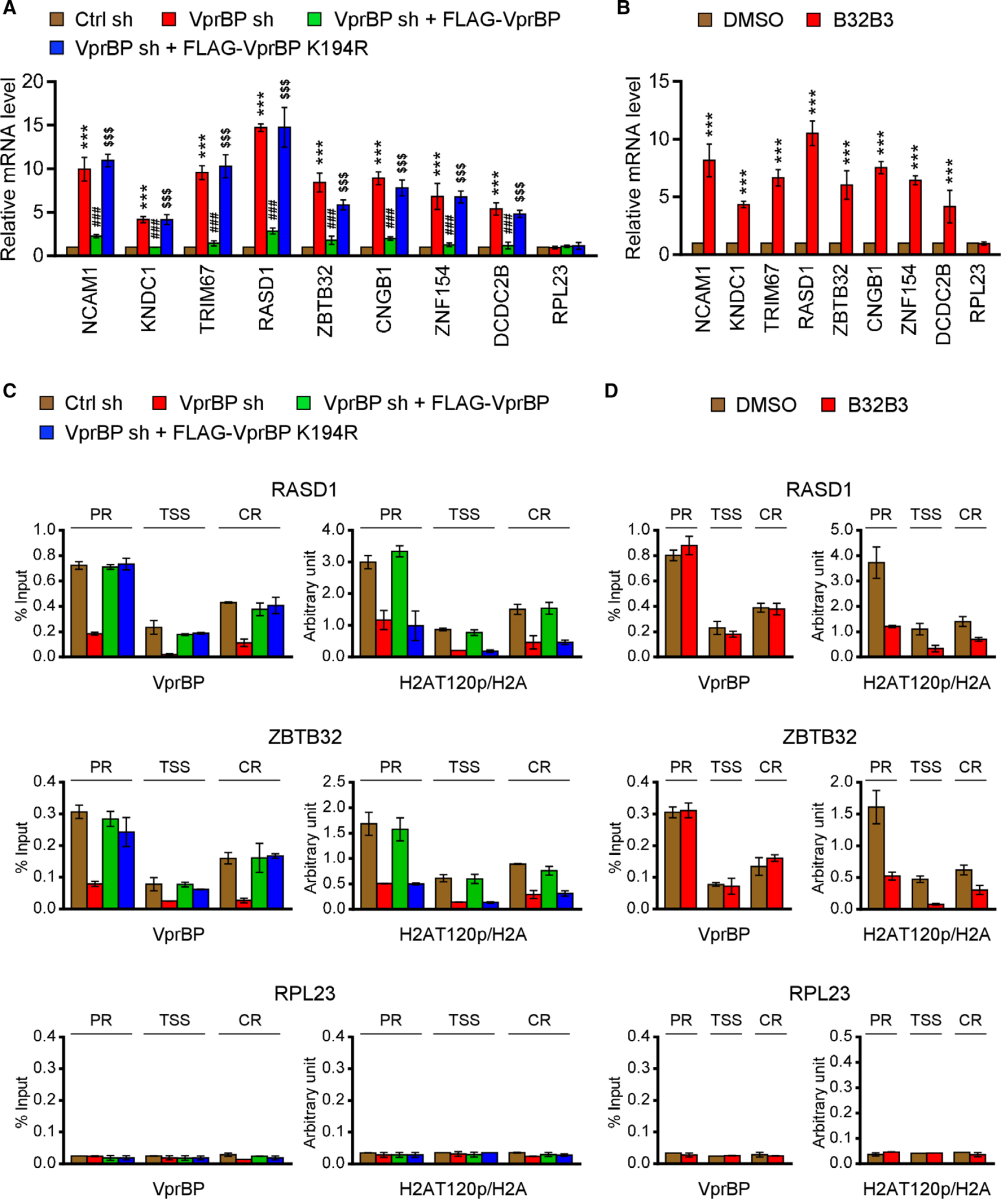
VprBP target genes are enriched for H2AT120 phosphorylation. (A) RNA samples were prepared from mock‐depleted control, VprBP‐depleted, or wild‐type/mutant VprBP‐transfected VprBP‐depleted SW620 cells and analyzed by RT–qPCR using primers listed in Table 1. All transcription levels were normalized to that of β‐actin. Data were expressed as mean ± SD (*N* = 3); *P* values were calculated using paired *t*‐tests. ***P < 0.001 versus Ctrl sh; ^###^ *P* < 0.001 versus VprBP sh; and ^$$$^ *P* < 0.001 versus Ctrl sh. (B) SW620 cells were treated with B32B3 inhibitor for 72 h, and VprBP target gene expression was analyzed by RT‐qPCR as in (A). Data were expressed as mean ± SD (*N* = 3); *P* values were calculated using paired *t*‐tests. ***P < 0.001 versus DMSO. (C) ChIP assays were performed in control, VprBP‐depleted, and VprBP‐complemented SW620 cells with VprBP, H2AT120p, and H2A antibodies as indicated. All ChIP DNAs were analyzed by real‐time PCR with six different primer pairs amplifying the promoters, transcription start sites, and coding regions of the two RASD1 (upper panel) and ZBTB32 (lower panel) genes. Primers used are listed in Table 2. Error bars denote the mean ± SD obtained from triplicate real‐time PCRs. (D) ChIP assays were performed as in (C), but using B32B3‐treated SW620 cells.

As an experiment to validate the RNA‐seq data, we next performed RT‐qPCR on the genes whose expression was enhanced upon VprBP depletion and which encode cell growth regulators. As summarized in Fig. 4A, RT‐qPCR analysis of the 8 upregulated genes showed that knockdown of VprBP caused ˜ 4‐ to 15‐fold increase in their mRNA levels. To further confirm our RNA‐seq results, we checked the rescue potential of ectopic VprBP. The expression of VprBP wild‐type in VprBP‐depleted SW620 cells fully restored the inactive states of target genes, whereas VprBP enzymatically dead mutant (K194R) failed to generate any detectable changes in their transcription rates (Fig. 4A). These results strongly argue that H2AT120p is the cause of the observed target gene inactivation. In all cases, the mRNA level of the non‐VprBP target gene RPL23 remains unchanged. In checking the functional importance of VprBP kinase activity toward H2AT120 in more direct fashion, we treated SW620 cells with B32B3, a potent and selective VprBP inhibitor, over a period of three days, and examined its influence on target gene transcription. Consistent with our previous study using prostate cancer cells, treating SW620 colon cancer cells with B32B3 at a concentration of 0.5 µm led to a marked increase in transcription levels of VprBP target genes (Fig. 4B). These results support our interpretation of VprBP knockdown data and confirm that VprBP shRNA specifically abrogated the effect of VprBP on suppressing target gene expression. In additional experiments using Caco2 colon cancer cells, knockdown and inhibition of VprBP generated similar effects on target gene transcription, again suggesting a major role for VprBP in keeping the target genes in an inactive state (Fig. S3A,B).

Whereas the above results suggested a role for VprBP in suppressing growth regulatory genes, we could not discriminate between direct and indirect effects of VprBP depletion on target genes in this analysis. We therefore used chromatin immunoprecipitation (ChIP) assays to investigate whether VprBP localizes along potential target genes. Among the VprBP‐regulated genes identified through RNA‐seq, RASD1 and ZBTB32 were selected for our analysis, because they are most significantly upregulated upon VprBP knockdown (Fig. 3) and because they are known to play critical roles in controlling cell proliferation, migration, and invasion [34, 35, 36, 37]. Crosslinked chromatin was isolated from control and VprBP‐depleted SW620 cells, and the precipitated DNA was amplified by quantitative PCR (qPCR) with three primer pairs targeting promoter region (PR), transcription start site (TSS), and coding region (CR) of the two target genes. We observed higher levels of VprBP and H2AT120p at the promoter region compared to the coding region and TSS, although the precipitation levels were slightly different between the two target genes (Fig. 4C). When ChIP experiments were performed in VprBP‐depleted cells, almost complete loss of H2AT120p ChIP signals was detected at target genes, strongly suggesting that VprBP is responsible for H2AT120p ChIP signals observed in these two target genes. Expressing ectopic VprBP wild‐type in VprBP‐depleted cells could largely override VprBP ChIP signal defects caused by VprBP knockdown and recover H2AT120p ChIP signals at the target genes (Fig. 4C). However, similar rescue analysis after expressing ectopic VprBP K194R mutant in VprBP‐depleted cells failed to show the recovery of H2AT120p ChIP signals at target genes, results indicative of the incapability of VprBP K194R mutant to generate H2AT120p. In the case of the RPL23 gene, which is not affected by VprBP knockdown, H2AT120p levels were very low and remained unchanged under all experimental conditions (Fig. 4C). In view of the observed dependency of H2AT120p on VprBP, it was also reasonable to assume that the reactivation of VprBP target genes in B32B3‐treated cells is accompanied by a reduction of H2AT120p at target genes. Indeed, treating SW620 colon cancer cells with B32B3 led to a significant reduction of H2AT120p levels at VprBP target genes (Fig. 4D). Analogous ChIP experiments were also conducted in Caco2 colon cancer cells, and similar data were obtained after VprBP knockdown and B32B3 treatment (Fig. S2C,D), again reinforcing the conclusion that VprBP is capable of localizing and establishing H2AT120p at target genes.

### 3.4 VprBP knockdown and inhibition attenuate the growth of colon cancer cells

**Fig. 5:**
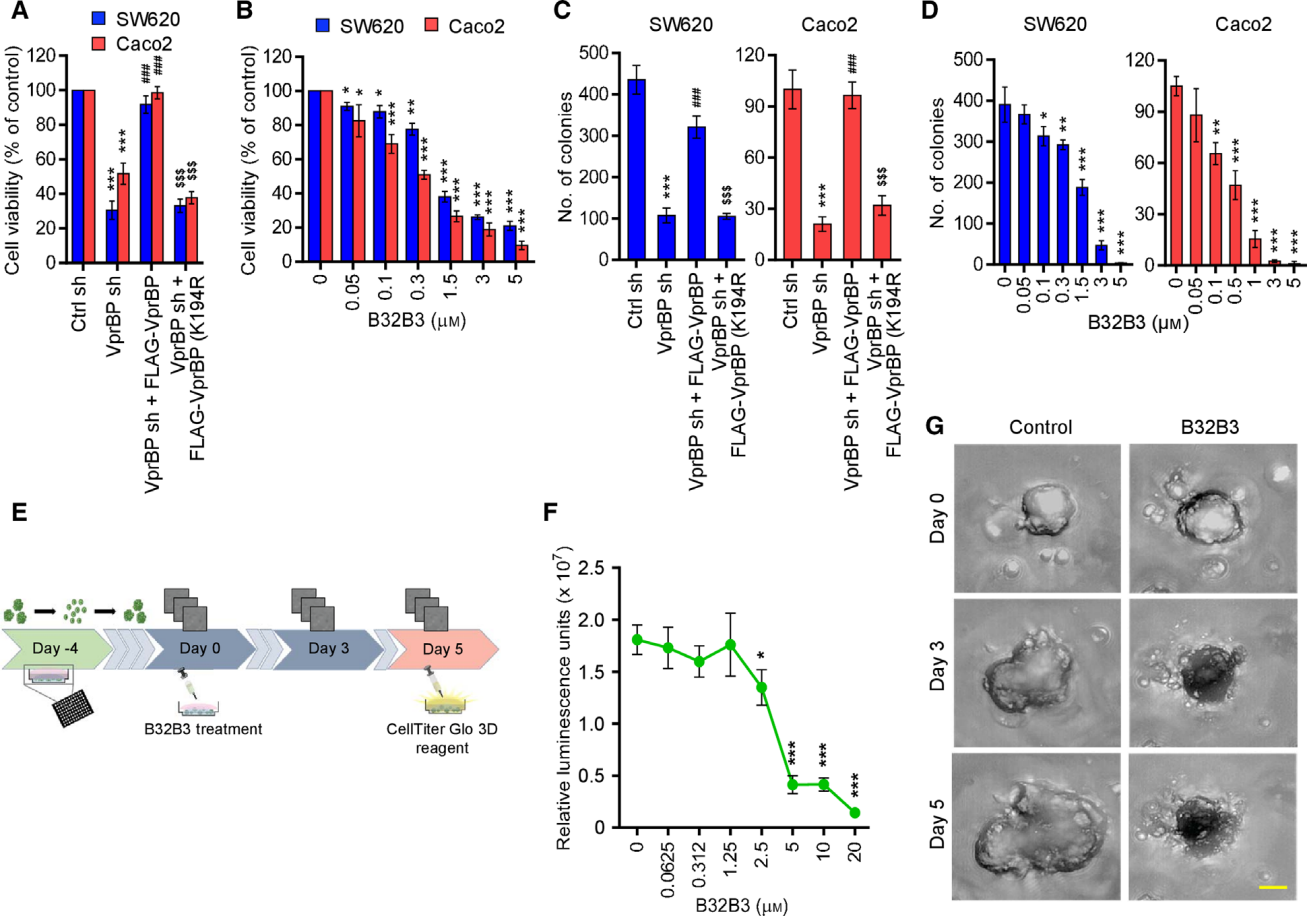
VprBP downregulation restricts colon cancer cell and organoid growth. (A) VprBP‐depleted SW620 (left panel) and Caco2 (right panel) cells were complemented with VprBP wild‐type or kinase‐dead mutant, and cell proliferation was assessed after 72 h of culture by WST‐1 assay. Results represent the mean ± SD of three experiments performed in triplicate; *P* values were calculated using paired *t*‐tests. ***P < 0.001 versus Ctrl sh; ^###^ *P* < 0.001 versus VprBP sh; and ^$$$^ *P* < 0.001 versus Ctrl sh. (B) SW620 and Caco2 cells were treated with increasing concentrations (0–20 µm) of B32B3 for 72 h, and their viability was measured by WST‐1 assay and is expressed as %. Data represent the mean ± SD of three independent experiments performed in triplicate; *P* values were calculated using paired *t*‐tests. *P < 0.05, **P < 0.01, and ***P < 0.001 versus 0 µm. (C) The indicated SW620 and Caco2 cells were allowed to form colonies for 2 weeks in 6‐well plates, stained with Crystal violet, and counted. Data represent the mean ± SD of three independent experiments in triplicate well; *P* values were calculated using paired *t*‐tests. ***P < 0.001 versus Ctrl sh; ^###^ *P* < 0.001 versus VprBP sh; and ^$$$^ *P* < 0.001 versus Ctrl sh. See also Fig. S3. (D) Colony formation assays were conducted as in C, but after treating with B32B3 at the indicated concentrations for 72 h. Data are representative of three independent experiments performed in triplicate and represent the mean ± SD; *P* values were calculated using paired *t*‐tests. *P < 0.05, **P < 0.01, and ***P < 0.001 versus 0 µm. (E) Schematic representation of B32B3 treatment of colon cancer patient organoids. Patient‐derived colon cancer organoids were digested to single cell, resuspended in a basement membrane extract, seeded in a 96‐well plate, and allowed to reform organoids for 4 days. Organoids were then treated with varying concentrations of B32B3 (0–20 µm) for five days and subjected to cell viability assay and microscopic imaging. (F) The CellTiter‐Glo luminescent cell viability assays were performed on B32B3‐treated organoids according to the manufacturer's instructions. Shown is the dose–response curve depicting relative luminescent units for each B32B3 concentration. Data are representative of three independent experiments performed in triplicate and represent the mean ± SD; *P* values were calculated using paired *t*‐tests. *P < 0.05, **P < 0.01, and ***P < 0.001 versus 0 µm. (G) Brightfield imaging was performed using a high content screening platform on days 0, 3, and 5 after treatment with 20 µm B32B3 or control treatment. Shown are the representative images of three independent experiments performed in triplicate. Scale bars correspond to 150 µm.

We next wanted to examine whether the above‐described effects of VprBP‐mediated H2AT120p on target genes reflect its functional property influencing the growth of colon cancer cells. Toward this end, we analyzed changes in cell viability in response to VprBP depletion in colon cancer cells after 72 h of culture. As summarized in Fig. 5A, our WST‐1 assays revealed a profound decrease in cell viability after stable knockdown of VprBP in SW620 and Caco2 cancer cells. To examine whether VprBP capacity to mediate H2AT120p is necessary for the observed effects, we also conducted rescue experiments. Unlike VprBP wild‐type, VprBP kinase‐dead mutant failed to recover the viability of VprBP‐depleted cancer cells, demonstrating that VprBP‐mediated H2AT120p is critical for VprBP function in promoting cell growth. Additionally, our colony formation assays also showed that knockdown of VprBP profoundly decreased the proliferation of SW620 and Caco2 cancer cells and that the expression of VprBP wild‐type, but not VprBP K194R mutant, restored cell proliferation rates (Fig. 5C and Fig. S4A). Since H2AT120p and VprBP target gene expression display a sharp decrease in B32B3‐treated cancer cells, we wondered whether the growth of colon cancer cells is also affected by B32B3 treatment. When examining this possibility, B32B3 caused a marked reduction in the viability of SW620 and Caco2 cells in a dose‐dependent fashion with an IC50 of approximately 0.9 and 0.3 µM, respectively (Fig. 5B). In our colony formation assays, it was also apparent that treatment with B32B3 decreased the clonogenic growth capacity of SW620 and Caco2 cells (Fig. 5D, Fig. S4B).

As a way to evaluate the growth inhibitory effects of B32B3 in a more physiological context, we next used patient‐derived organoid (PDO) models (Fig. 5E). PDOs recapitulate the histologic, genetic, and molecular features of the tumors from which they are derived and show high genome stability. They are therefore a suitable complement to our cell line‐based assays for more physiological evaluation of antitumor efficacy of B32B3. To conduct our experiments, we first generated colonic cancer PDO cultures and confirmed their spherical morphologies with variably sized diameters. Then, we evaluated the response of PDO cultures to a 5‐day treatment with B32B3 at doses ranging from 0.0625 to 20 µm. As evident from our luminescent cell viability assays, B32B3 treatment resulted in a significant reduction in PDO growth in a dose‐dependent manner with a steep drop between 1.25 µm and 5 µm (Fig. 5F). Consistent with these observations, phase contrast images taken from untreated PDO samples showed a fivefold increase in size after 5 days of culture, but treatment with B32B3 (20 µm) efficiently rescued enlarged organoid sizes and disorganized organoid architectures (Fig. 5G).

### 3.5 VprBP inhibitor has antitumor activity in preclinical colon cancer models

**Fig. 6:**
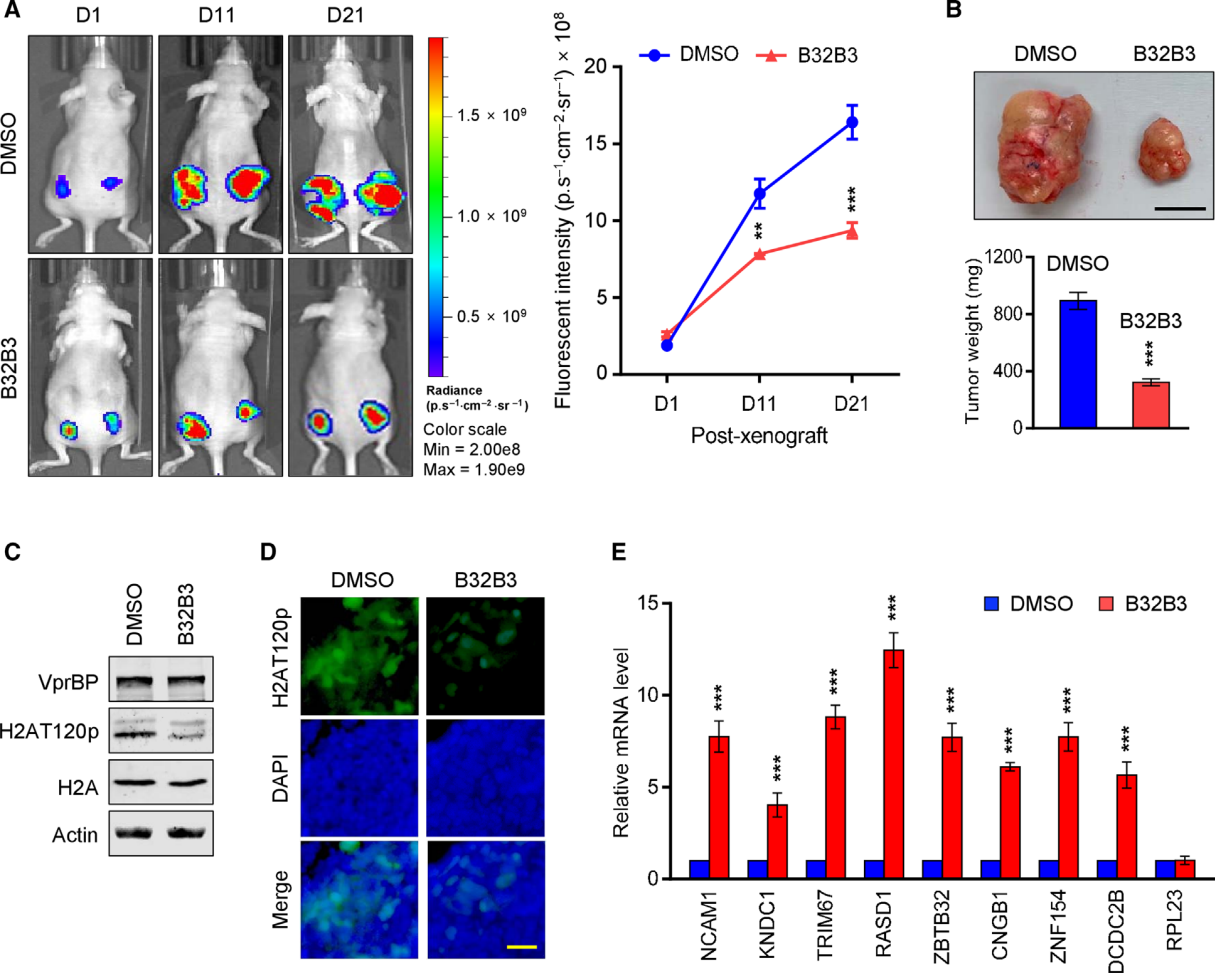
VprBP inhibitor suppresses tumor growth *in vivo*. (A) Colon tumor xenografts were established by subcutaneously injecting EGFP‐expressing SW620 cells into the right and left hind legs of mice. Mice were then treated with B32B3 (5 mg/kg) or an equivalent volume of vehicle twice weekly for 21 days, and SW620 xenograft tumor growth was monitored by checking fluorescent signals with an *in vivo* imaging system (IVIS). GFP‐fluorescence images (left) and fluorescence intensity quantification (right) after 1, 11, and 21 days of B32B3 treatments are shown. Data are represented as the mean ± SEM (*N* = 8); *P* values were calculated using paired *t*‐tests. **P < 0.01 and ***P < 0.001 versus DMSO. (B) Mice were sacrificed on day 21 after the last B32B3 treatment, and tumor xenografts were surgically excised and photographed (upper panel, scale‐1 cm). Tumor weights were also measured and expressed in milligrams (lower panel). Data are represented as the mean ± SEM (*N* = 8); *P* values were calculated using paired *t*‐tests. ***P < 0.001 versus DMSO. (C, D) Mice were sacrificed at the end of the treatment schedule (day 21), and subcutaneous tumors were extracted and analyzed by western blotting (C) and immunostained (D) with the indicated antibodies. Scale bars correspond to 5 µm. (E) On day 21 after mice received the last treatment, relative expression levels of VprBP target genes in vehicle‐treated and B32B3‐treated SW620 xenografts were determined by RT‐qPCR. Data represent the mean ± SD (*N* = 8); *P* values were calculated using paired *t*‐tests. ***P < 0.001 versus DMSO.

The data presented above argue persuasively that B32B3 treatment interferes with VprBP‐mediated H2AT120p and colon cancer cell growth. However, it is not clear whether B32B3 is also effective in inhibiting colonic tumor growth *in vivo*. If VprBP exerts its oncogenic effects mainly by catalyzing H2AT120p, then we can predict that B32B3 treatment should efficiently modulate colonic tumor development and progression. To address this question, we chose to use a cell line‐derived xenograft model expressing enhanced green fluorescent protein (eGFP). Consequently, SW620 cells were transfected with a lentiviral vector encoding the eGFP, subcutaneously injected into both flanks of nude mice, and treated with intraperitoneal injections of B32B3 at a dose of 5 mg·kg^−1^ twice a week over 21 days. As shown in Fig. 6A, our fluorescence imaging at days 1, 11, and 21 demonstrated that treatment with B32B3 significantly impaired the proliferative ability of SW620 xenograft tumors. After 3 weeks of B32B3 treatment, mice showed no significant changes in body weight (Fig. S5). B32B3, thus, must have inhibitory effects on the growth of SW620 xenograft, specifically targeting SW620 cancer cells in mice.

In additional analysis, we sacrificed mice on day 21 and harvested the xenograft tumors to determine to what extent B32B3 treatment affected the level of H2AT120p. Similar to fluorescence imaging analyses reported above, B32B3 treatment decreased tumor weight by ˜ 70% (Fig. 6B). When we conducted western blotting with SW620 xenograft lysates, we detected high levels of H2AT120p in mock‐treated control xenograft tumors. However, the observed levels of H2AT120p were decreased to nearly 30% after B32B3 treatment, strongly supporting that B32B3 inhibits xenograft tumor growth in an H2AT120p‐selective manner (Fig. 6C). Similar results were obtained when control and B32B3‐treated cells were immunostained with H2AT120p antibody and compared under a fluorescent microscope (Fig. 6D). Considering that H2AT120p is essential for VprBP transrepression, we also examined the effects of B32B3 on VprBP target gene expression in the xenograft tumors. We found that B32B3 treatment resulted in, albeit to a varying extent, higher expression of VprBP target genes in SW620 xenograft tumors (Fig. 6E). These and the above noted results overall support the idea that B32B3 treatment reactivates VprBP target gene expression and inhibits colonic tumor growth by altering H2AT120p levels.

## 4 Discussion

Most studies that have examined the role of VprBP in cancer have focused on its role as a substrate recognition component of E3 ubiquitin ligase complexes [7, 38]. However, our recent finding that VprBP has an intrinsic kinase activity targeting H2AT120 offered the new aspect that VprBP is linked to epigenetic gene regulation in the context of chromatin. In further support of VprBP function in oncogenic signaling pathway, we showed that VprBP is highly expressed in prostate cancer cells and inactivates growth‐controlling genes through H2AT120p. Also, our systematic screening of small‐molecule library identified B32B3 as a selective inhibitor, blocks VprBP kinase activity toward H2AT120, and suppresses prostate cancer growth *in vivo* [11]. Although our investigation uncovered a new function for VprBP as a histone kinase driving prostate cancer, it remains unclear whether VprBP plays a similar role in the development of other types of cancer.

In this report, we describe systematic analyses of VprBP and show that VprBP‐mediated H2AT120p is a major epigenetic event to drive oncogenic gene silencing in colon cancer development. An initial finding from our investigation is that overexpression of VprBP is closely related to elevated levels of H2AT120p in colon cancer cells. These results raise the possibility that VprBP‐mediated H2AT120p may contribute toward inactivating the network of growth regulatory genes during colon carcinogenesis. Supporting this idea, our genome‐wide analysis demonstrated that genes targeted by VprBP in colon cancer cells are heavily enriched in genes whose expression increases cell growth and proliferation. The majority of these genes are downregulated through H2AT120p, since VprBP wild‐type, but not VprBP kinase‐dead mutant, can restore H2AT120p and target gene silencing in VprBP‐depleted cells.

These results are in line with our previous reports indicating that VprBP‐mediated H2AT120p establishes and maintains the inactive state of genes essential for normal cell growth and proliferation in prostate cancer cells. Our current study, however, allowed us to identify only a subset of VprBP targets whose expression is deregulated in prostate cancer cells. We speculate that the prostate and colon cancer cell lines used in our studies have unique, cell type‐specific chromatin accessibility patterns that may influence the efficiency of VprBP‐mediated H2AT120p and gene silencing. It is also plausible that VprBP‐mediated H2AT120p could regulate different sets of targets in different types of cancer, namely by crosstalk with different functional partners capable of regulating specific genomic signals and regions; additional studies will broaden the spectrum of potential VprBP targets for specific cancer types.

Our demonstration of the striking correlation between the levels of VprBP and H2AT120p at target genes argues strongly that VprBP is a prominent regulator of H2AT120p in colon cancer cells. In agreement with these observations, our cell viability and colony formation experiments show that VprBP and its kinase activity are indeed essential for the proliferative potential of colon cancer cells. Another approach that we employed in our investigation was to treat cells with B32B3 which is a highly potent and selective VprBP inhibitor. This study confirmed that B32B3 treatment modulates the expression of growth inhibitory genes and the ability of cancer cell to proliferate *in vitro*. Based on these data, we predicted that colon cancer cells would grow fast as xenografts in nude mice, while B32B3‐treated cells would grow slowly. *In vivo* experiments supported this hypothesis and confirmed the importance of VprBP in colon tumorigenesis. Additionally, B32B3 treatment generated an almost complete disappearance of H2AT120p and a reactivation of VprBP target genes in colonic tumor xenografts. We also observed that, in contrast to SW620 and Caco2 cells overexpressing VprBP, treatment of T84 colon cancer cells displaying only low VprBP expression (Fig. 1C) with B32B3 failed to prevent the ability of the cells to grow (data not shown). These data therefore suggest that B32B3 is specifically targeting VprBP kinase activity and that the observed suppression of cancer cell growth is generated from B32B3 effects on VprBP‐mediated H2AT120p and downstream gene silencing pathway. The observed selectivity of B32B3 is also important, because new cancer drug development efforts aimed at identifying epigenetic misregulations must consider not only the effect of target inhibition on tumor cells, but also its impact on normal cell physiology.

Our demonstration that VprBP‐induced colon tumor pathogenesis occurs dependently of H2AT120p is also important, given recent reports linking altered global levels of histone modifications to a cancer phenotype in colon cancers [39, 40]. Moreover, aberrant expression of some histone‐modifying factors has been postulated to promote the proliferation and malignant state of colon cancer cells [41, 42, 43, 44, 45]. To our knowledge, however, altered histone modification per se has not been shown to directly affect gene transcription and oncogenic potential in the colon cancer development process. Here, we provide ample evidence to confirm that VprBP drives oncogenic gene silencing through H2AT120p in colon cancer cells. Perhaps more important are our results highlighting H2AT120p as a new histone mark in the development of colon cancer. As a potential therapeutic benefit of using inhibitors targeting histone‐modifying factors has not been well explored, our results also suggest that pharmacological inhibition of VprBP kinase activity is a useful tool to regulate the growth and proliferation of colon cancer cells. Further, establishing H2AT120p to alter gene transcription and cell growth is unique among prior reports of epigenetic mechanisms underlying colon cancer and is of particular interest because of VprBP to be the only kinase responsible for the modification. The notion that VprBP‐mediated H2AT120p is cancer cell‐specific in regulating gene transcription also offers the possibility that similar mechanisms may apply to other cancer types. Discerning whether this is the case and how they may induce altered cell characteristics will help define whether VprBP‐mediated H2AT120p represents a general driving force behind cancer development. Additionally, as done with other histone modifications [40], the findings described here also raised the possibility of using H2AT120p as a new biomarker for colon cancer and exploiting the dynamic nature of epigenetic changes to potentially modulate responses to cancer treatments.

## 5 Conclusions

In conclusion, our work shows that VprBP is overexpressed and exerts a protumorigenic function in colonic carcinogenesis by triggering H2AT120p and epigenetic gene silencing. Because the VprBP inhibitor B32B3 potently suppresses the growth of colon cancer cells, more detailed evaluation of its efficacy and combination use with other cancer grow inhibitors, for example, DNMT inhibitors, will be of particular interest. Many of the enzymes that modify histones have nonhistone substrates that are also involved in inappropriate gene regulation in a broad range of cancer types. Thus, it is tempting to speculate that nuclear nonhistone proteins could also be phosphorylated by VprBP, which might play an additional role in establishing a transcriptionally incompetent chromatin and gene silencing in colon cancer cells. In this context, it will be of great interest to determine the full spectrum of VprBP function in targeting H2A and nonhistone proteins as well as inducing oncogenic transformation at a molecular level.

## Conflict of interest

The authors declare no conflict of interest.

## Author contributions

NBG and WA conceived and designed the study. NBG, SK, ES, SK, and YS performed the experiments with contributions of GS, HJL, SMM, and WA. NBG, SK, ES, YS, and WA analyzed the data. SKR analyzed the RNA‐seq data. NBG and WA wrote the manuscript. All authors read and approved the final manuscript.

### Peer Review

The peer review history for this article is available at https://publons.com/publon/10.1002/1878‐0261.13068.

## Supporting information

**Fig. S1**. (A) The Kaplan–Meier overall survival analysis of 440 colon cancer patients based on VprBP expression status using the OncoLnc online tool (http://www.oncolnc.org/). (B) Violin plots of VprBP expression in different clinical stages of Colon adenocarcinoma (COAD). Gene Expression Profiling Interactive Analysis (GEPIA) online tool (http://gepia.cancer‐pku.cn/) was used for the analysis.
**Fig. S2**. A heatmap shows 1,649 upregulated genes and 895 downregulated genes upon VprBP knockdown.
**Fig. S3**. RT‐qPCR (A and B) and ChIP‐qPCR (C and D) analyses were performed as in Figure 4, but using VprBP‐depleted/rescued (A and C) or B32B3‐treated (B and D) Caco2 cells.
**Fig. S4**. Shown are representative pictures of SW620/Caco2 cell colony formation upon VprBP knockdown/rescue (A) or B32B3 treatment (B). See also Figure 5B and D.
**Fig. S5**. Body weights of vehicle or B32B3‐treated mice were monitored every three days after the first treatment. Mean body weights (g) ± SEM are shown.

## Data Availability

The data that support the findings of this study are available in NCBI's Gene Expression Omnibus and are accessible through https://www.ncbi.nlm.nih.gov/geo/query/acc.cgi?acc=[GSE180282], GEO Series accession number [GSE180282].
