# Supplementary material for: VprBP directs epigenetic gene silencing through histone H2A phosphorylation in colon cancer
Source: Mol Oncol. 2021 Aug 8;15(10):2801–17. doi: 10.1002/1878-0261.13068 (PMC8486565; doi:10.1002/1878-0261.13068)
Supplement: Supplementary file 1 — Fig. S1. (A) The Kaplan–Meier overall survival analysis of 440 colon cancer patients based on VprBP expression status using the OncoLnc online tool (http://www.oncolnc.org/). (B) Violin plots of VprBP expression in different clinical stages of Colon adenocarcinoma (COAD). Gene Expression Profiling Interactive Analysis (GEPIA) online tool (http://gepia.cancer‐pku.cn/) was used for the analysis. Fig. S2. A heatmap shows 1,649 upregulated genes and 895 downregulated genes upon VprBP knockdown. Fig. S3. RT‐qPCR (A and B) and ChIP‐qPCR (C and D) analyses were performed as in Figure 4, but using VprBP‐depleted/rescued (A and C) or B32B3‐treated (B and D) Caco2 cells. Fig. S4. Shown are representative pictures of SW620/Caco2 cell colony formation upon VprBP knockdown/rescue (A) or B32B3 treatment (B). See also Figure 5B and D. Fig. S5. Body weights of vehicle or B32B3‐treated mice were monitored every three days after the first treatment. Mean body weights (g) ± SEM are shown. [file MOL2-15-2801-s001.pdf]

Supplementary Figure 1

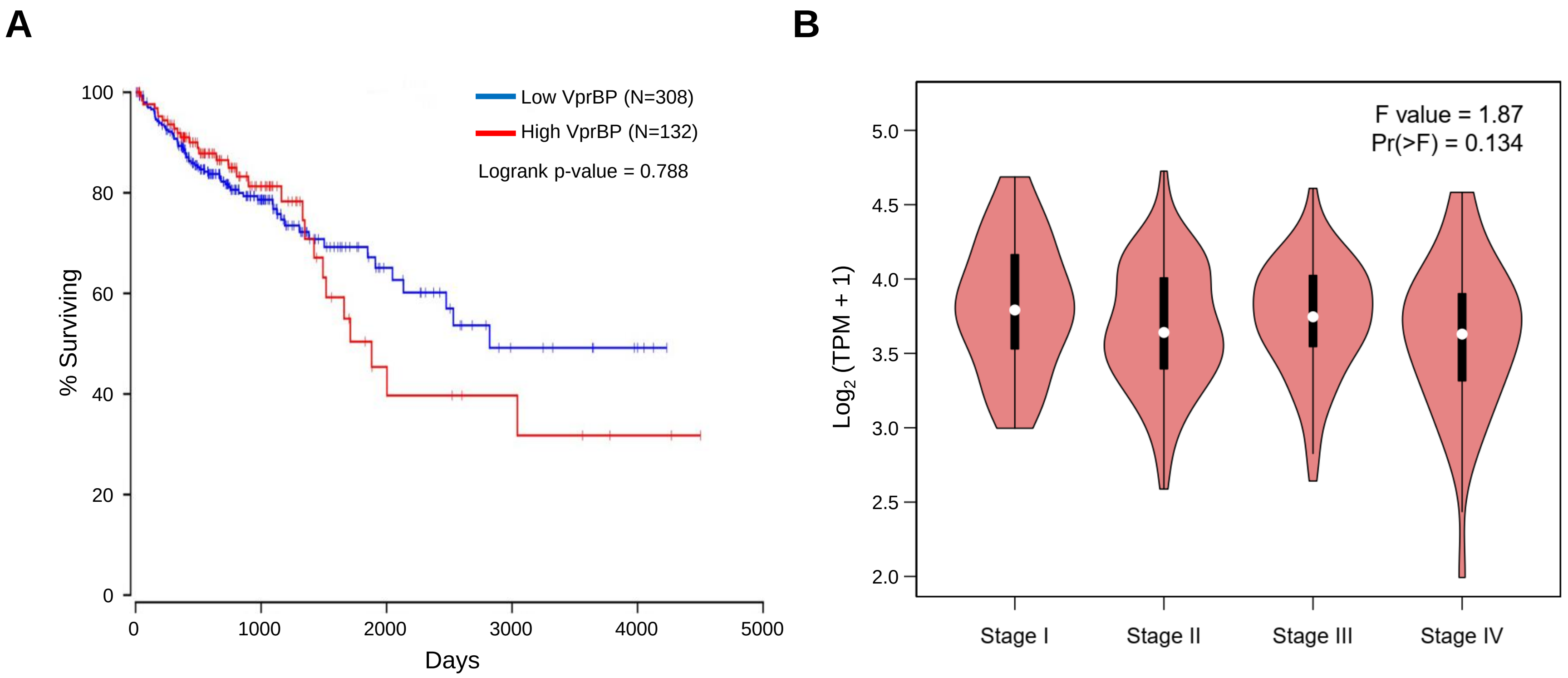

Supplementary Figure 1.
(A) The Kaplan–Meier overall survival analysis of 440 colon cancer patients based on VprBP expression status using the OncoLnc online tool (http://www.oncolnc.org/).
(B) Violin plots of VprBP expression in different clinical stages of Colon adenocarcinoma (COAD). Gene Expression Profiling Interactive Analysis (GEPIA) online tool (http://gepia.cancer-pku.cn/) was used for the analysis.

Supplementary Figure 2

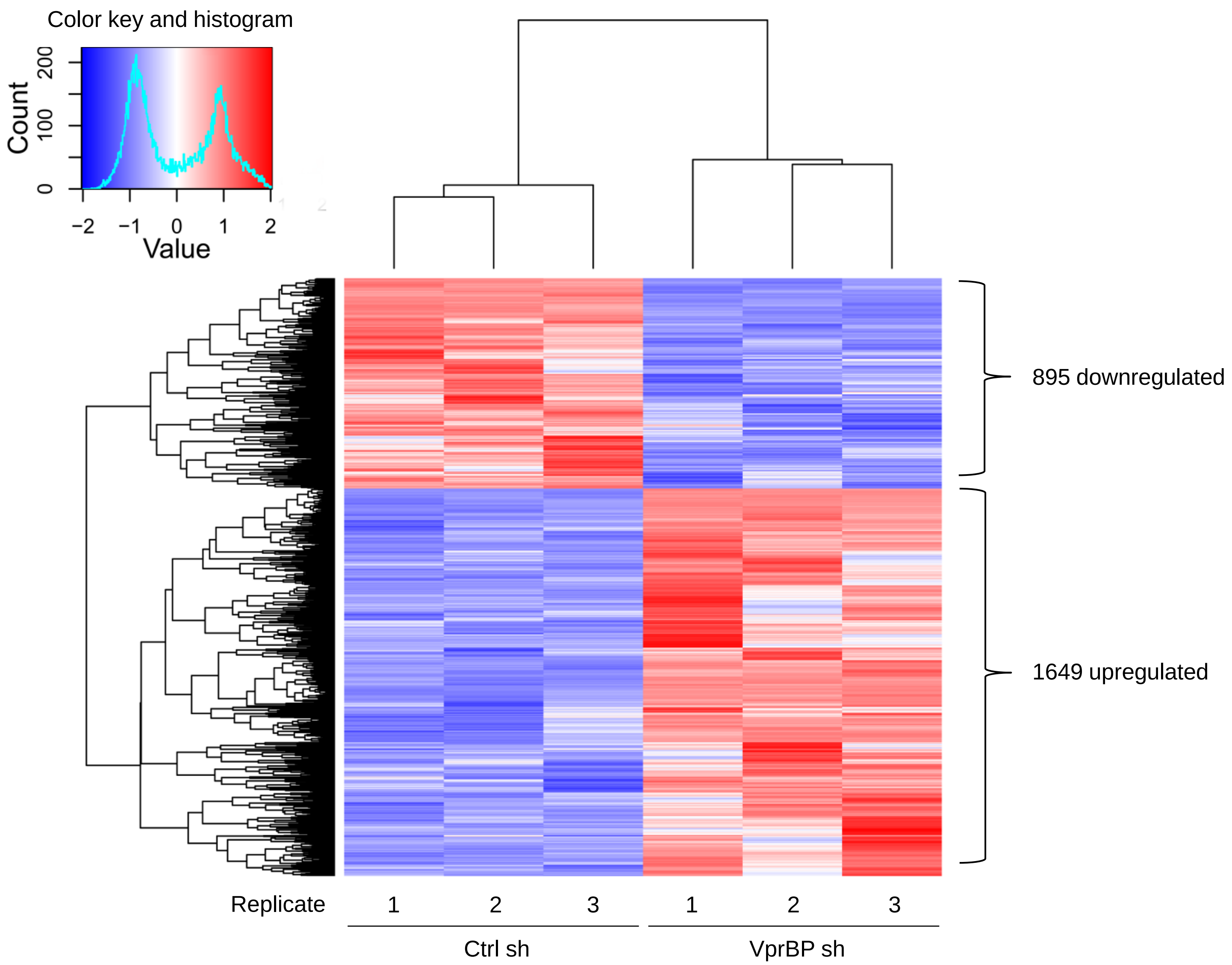

Supplementary Figure 2.
A heatmap shows 1,649 upregulated genes and 895 downregulated genes upon VprBP knockdown. Normalized gene expression levels (Z-scores) are plotted. Color key indicates for Z score (blue: low expression, red: high expression). Histogram indicates the number of genes that belong to each category.

Supplementary Figure 3

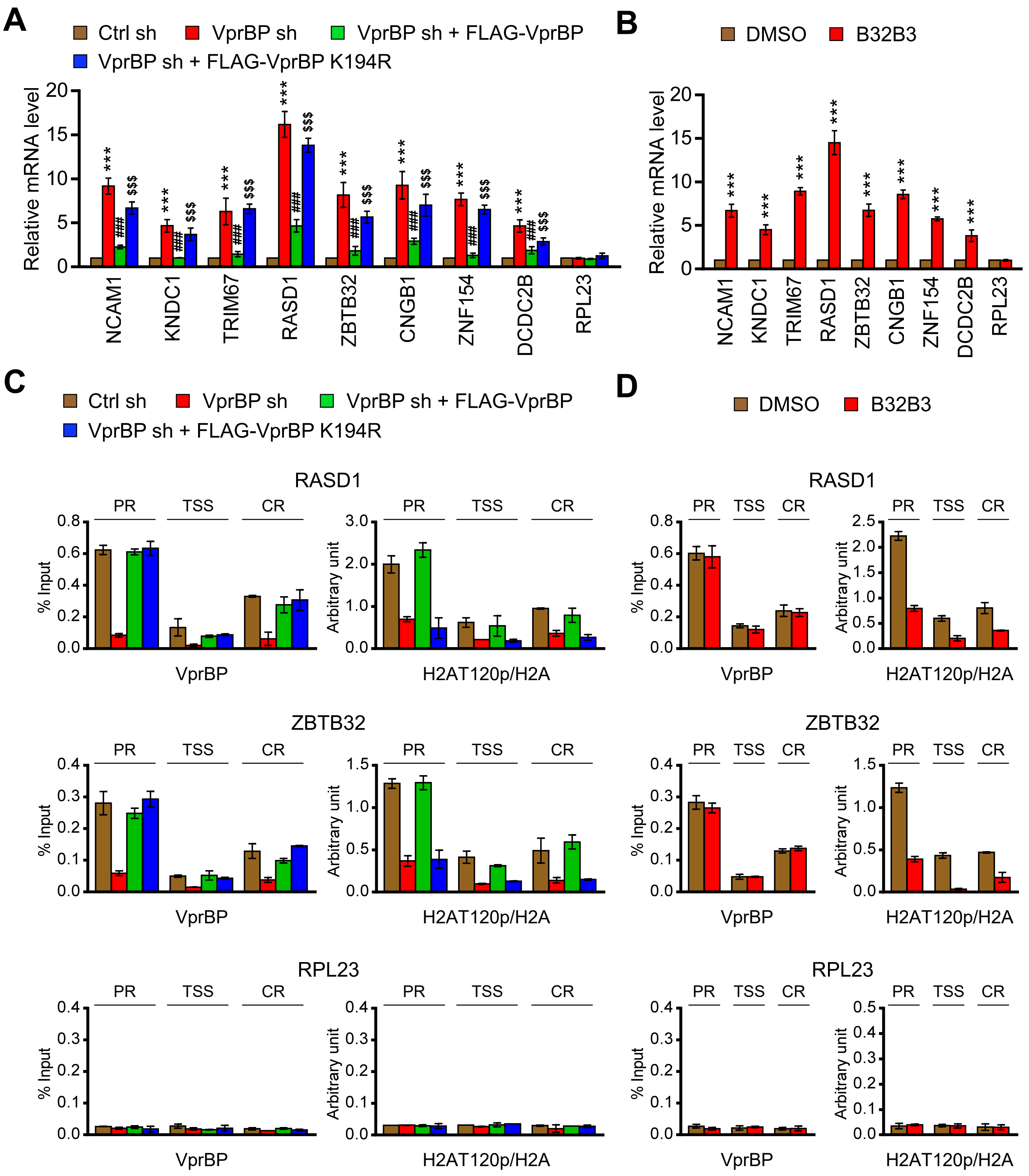

Supplementary Figure 3.
RT-qPCR (A and B) and ChIP-qPCR (C and D) analyses were performed as in Figure 4, but using VprBP-depleted/rescued (A and C) or B32B3-treated (B and D) Caco2 cells.
RT-qPCR data were expressed as mean ± S.D. (N=3). For (A): ***P<0.001 *versus* Ctrl sh; ###P<0.001 *versus* VprBP sh; $$$P<0.001 *versus* Ctrl sh. For (B): ***P<0.001 *versus* DMSO.

Supplementary Figure 4

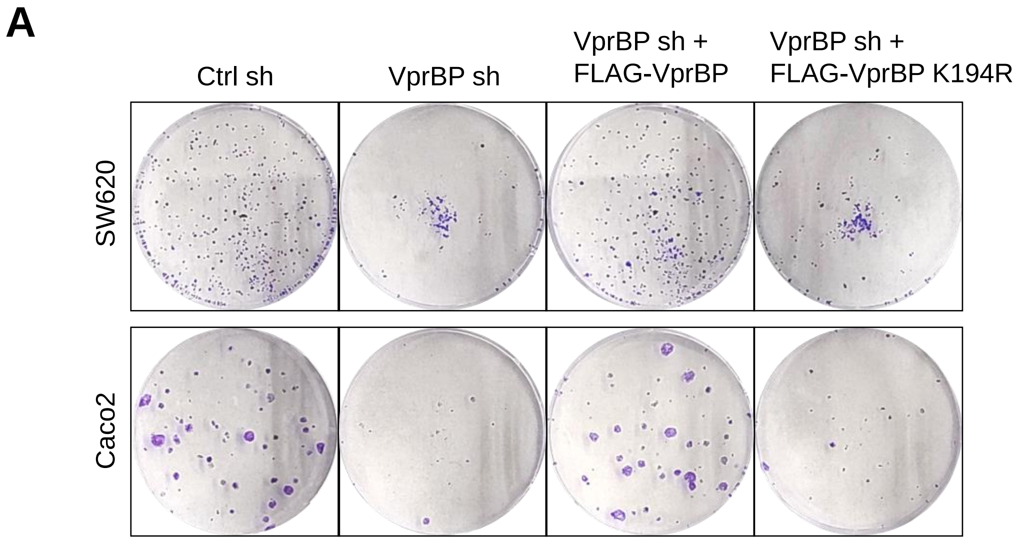

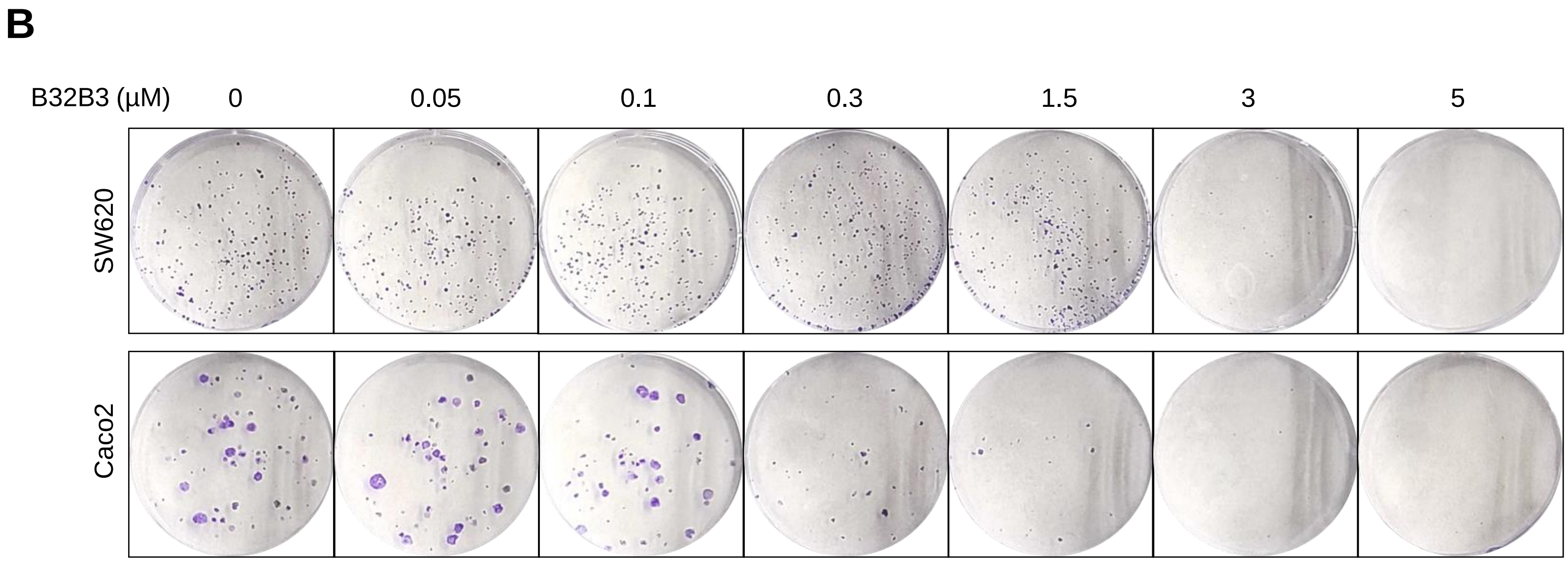

Supplementary Figure 4.
Shown are representative pictures of SW620/Caco2 cell colony formation upon VprBP knockdown/rescue (A) or B32B3 treatment (B). See also Figure 5B and D.

Supplementary Figure 5

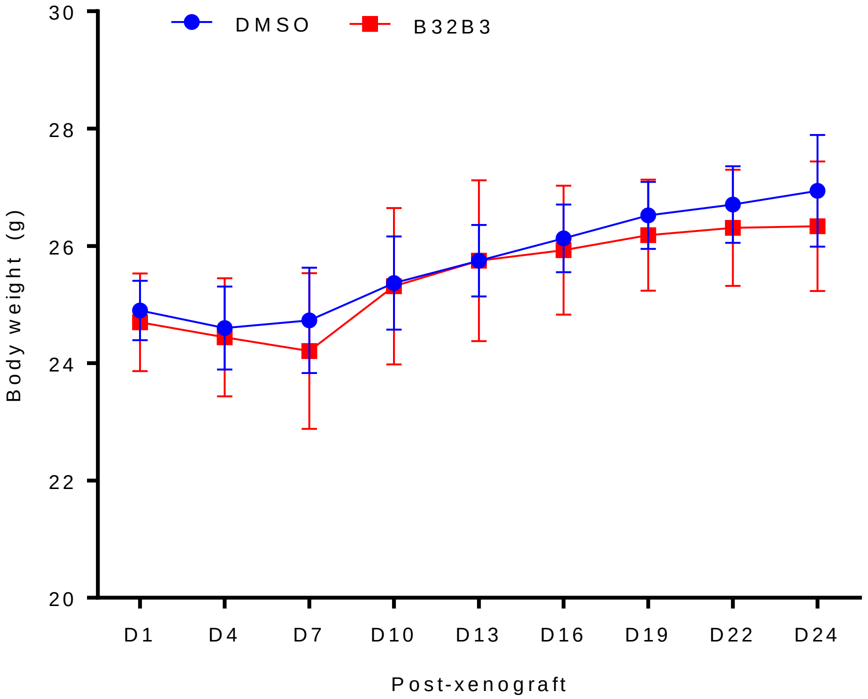

Supplementary Figure 5.
Body weights of vehicle or B32B3-treated mice were monitored every three days after the first treatment. Mean body weights (g) ± SEM are shown.
